# Developing an evaluation system for creativity courses in design disciplines oriented to education for sustainable development: an integrated application of AHP-entropy weighting and FCE models

**DOI:** 10.3389/fpsyg.2026.1709780

**Published:** 2026-04-30

**Authors:** Xuebing Fang, Huaixi Xu, Xing Yang, Niu Liu, Liujun Xu, Die Hu, Lei Huang, Heng Jiang, Zheng Fang

**Affiliations:** 1 School of Art and Design, Huainan Normal University, Huainan, China; 2 School of Mechanical and Electrical Engineering, Anhui Jianzhu University, Hefei, China; 3 School of Psychology, HSE University, Moscow, Russia

**Keywords:** education for sustainable development (ESD), creativity courses, design education, AHP-EWM-FCE, course evaluation system

## Abstract

**Introduction:**

Education for sustainable development (ESD) has become a key focus in higher education transformation. Design disciplines play a unique role in cultivating students' creativity to address complex social and ecological challenges, yet current course evaluation systems remain disconnected from ESD values and lack quantitative tools.

**Methods:**

This study constructed an ESD-oriented course evaluation system for creativity courses in design disciplines, covering four dimensions: course philosophy, course content, course teaching, and course evaluation. It integrated the analytic hierarchy process (AHP) and entropy weight method (EWM) for combined weighting, and adopted fuzzy comprehensive evaluation (FCE) for empirical assessment at a regional university in China.

**Results:**

The weights of course philosophy (0.3801) and course evaluation (0.3239) were the highest among the four dimensions. The empirical case showed an overall score of 70.35, with course evaluation scoring highest (76.50) and course philosophy lowest (62.78), revealing a structural contradiction between instrumental rationality and value rationality.

**Discussion:**

The study verified the feasibility of the AHP-EWM-FCE model and proposed a reverse evaluation paradigm centered on philosophy and evaluation. The findings provide a quantitative tool and improvement pathway for ESD integration and creativity cultivation in design education.

## 1 Introduction

In the context of globalization and digitalization in the 21st century, education for sustainable development (ESD) has become a major driving force in social transformation and higher education reform. UNESCO (2015, 2021) clearly stated in the 2030 Agenda for Sustainable Development and Reimagining Our Futures Together that ESD is not only a key pathway to achieving the Sustainable Development Goals (SDGs), but also a core mechanism for cultivating the capacities that future citizens need to address complex and uncertain challenges. This suggests that education in the era of sustainability must move beyond knowledge transmission and place greater emphasis on Critical Thinking, innovation, and social participation.

At present, generative artificial intelligence (GenAI) is reshaping design productivity. As an interdisciplinary field that links technology, engineering, art, and social development, design disciplines are shifting their educational goals from traditional aesthetic form-giving to ecological symbiosis, with a growing emphasis on the deep integration of creativity and sustainability-related abilities (Schulz et al., 2021). However, current Design Education systems face multiple challenges. Social change requires designers to become interdisciplinary problem solvers, while digital technologies such as VR, AR, and GenAI are reshaping learning models, yet they lack adequate curricular and faculty support (Meirbekov et al., 2022). In China, especially in regional universities, curricular systems and evaluation mechanisms in design education have not kept pace with these changes. Many schools of art and design still maintain teacher-centered traditions, and course evaluation remains strongly focused on outcomes and intuitive judgment (Xu and Liu, 2024). As a result, students' actual performance in interdisciplinary collaboration, Ecological Ethics Awareness, and sustainable innovation practice is not effectively captured.

Creativity courses are central to design education. Their essential function is to stimulate innovative thinking, thereby promoting problem discovery and the generation of new solutions. Yet, from the perspective of disciplinary development, Chinese design education has long been shaped by the traditions of painting and arts-and-crafts instruction and has generally prioritized skills over evaluation (Zhu, 2015). This evaluation logic, characterized by an emphasis on result scoring and a neglect of value orientation (Darbellay, 2024; Zhang et al., 2024), is seriously disconnected from the values-abilities-actions (VAA) Triad advocated by ESD. It not only constrains the deeper development of students' innovative thinking, but may also weaken the social value of design education when responding to complex socio-ecological challenges.

From the perspective of course development, the evaluative function of the curriculum has not yet been fully activated. Evaluation should not merely serve as an external judgment of learning outcomes; it should also function as the central coordinator linking course philosophy, the teaching process, and learning outcomes. Effective course evaluation can connect course objectives, content, implementation, and outcomes, provide a scientific basis for teaching improvement (Sconce and Howard, 1994), and guide teaching away from the simple transmission of skills toward the construction of literacy. However, existing evaluation systems remain insufficient for addressing the complexity of design creativity. Three major research gaps are particularly evident. First, creativity evaluation in design education has not yet been deeply integrated with ESD, and there is still no systematic approach to measuring sustainable creativity. Second, in design-related fields, ESD-oriented evaluation lacks a quantitative evaluation tool that can accommodate both qualitative meaning and quantitative analysis. Third, the traditional linear paradigm of “objectives-content-evaluation” does not fit the inquiry-based and interdisciplinary demands of creativity development and urgently needs to shift toward a Reverse Design Paradigm centered on educational philosophy.

**Figure 1:**
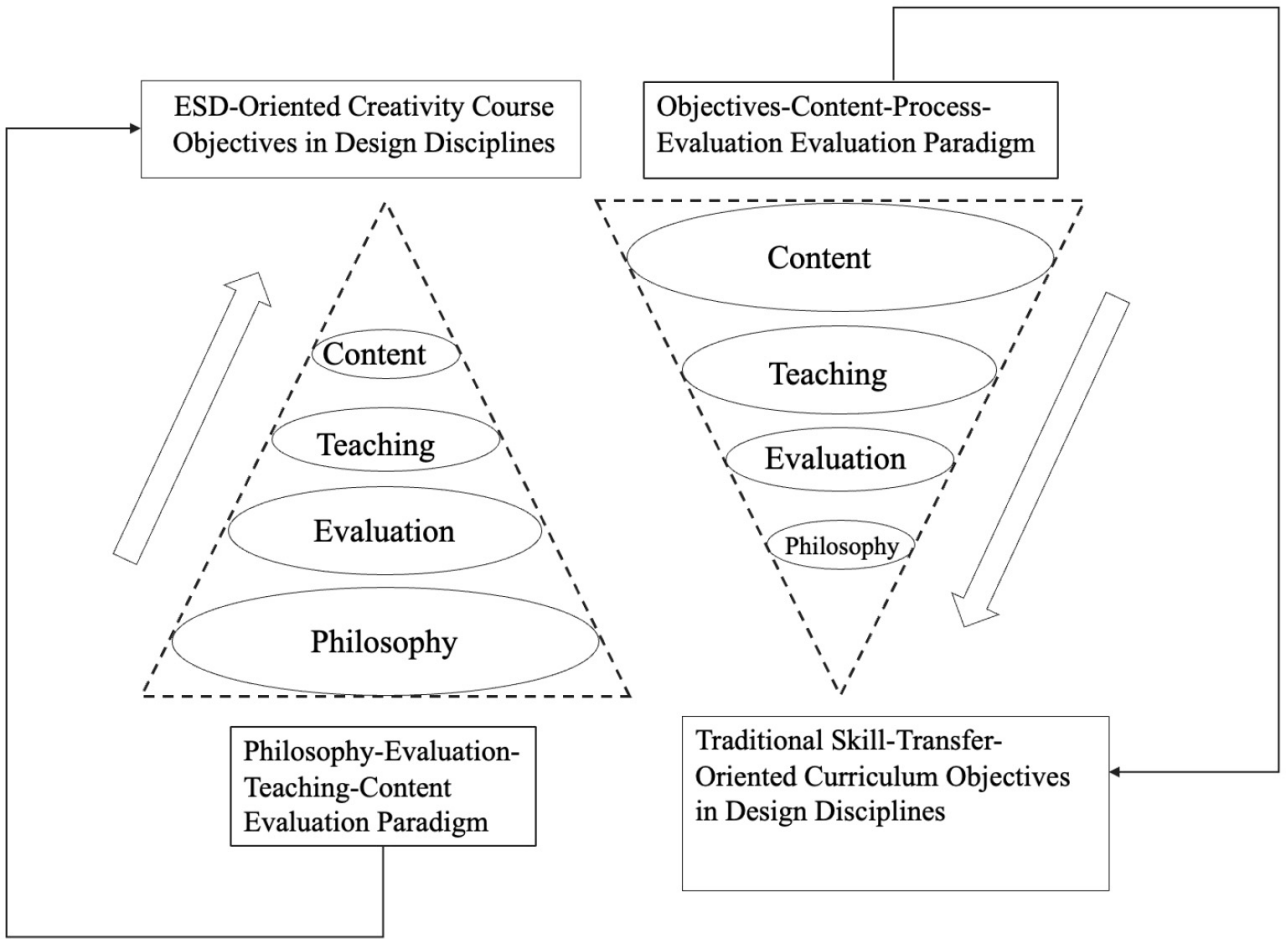
The logical shift from the traditional course evaluation paradigm to the evaluation paradigm proposed in this study.

Against this background, this study aimed to construct a course evaluation system for creativity courses in design disciplines oriented to ESD. First, based on literature analysis and expert consultation, it identified evaluation indicators across four dimensions: philosophy, content, teaching, and evaluation. Second, it integrated AHP and EWM to generate the combined weighting (AHP-EWM), thereby balancing expert judgment and the distribution of objective data. Finally, fuzzy comprehensive evaluation (FCE) was introduced to conduct an empirical assessment using a representative regional university in China (“H” University) as an exploratory case. In re-examining the logic of course evaluation, this study found that the traditional linear model of “objectives-content-process-evaluation” often lagged behind teaching practice. Such a model easily leads to an emphasis on outcomes while neglecting philosophy, reducing evaluation to a retrospective summary. Conversely, this study proposed a reconstruction based on the reverse sequence of “philosophy-evaluation-teaching-content.” As shown in Figure 1, course philosophy is at the core of the evaluation system, and evaluation mechanisms drive systematic renewal of teaching methods and content. This shift is intended to respond to ESD's deep demand for the student-centered principle and interdisciplinary inquiry by transforming evaluation from a judgment tool into a lever for educational transformation. Through this reverse reconstruction, the study seeks to identify the structural contradictions embedded in the current transformation of design education more precisely and to provide a quantitative basis and improvement pathway for moving from isolated skill training to the development of creativity and sustainability literacy.

## 2 Literature review

### 2.1 Education for sustainable development and the reconstruction of higher education capacities

The term “sustainability” originated in environmental research in German forestry in the 1980s (Klarin, 2018). Our common future provided the classic definition of sustainable development, stating that development should meet present needs without compromising the ability of future generations to meet their own needs (World Commission on Environment and Development, 1987; Evans, 2010). This definition implies a shift in value judgment from anthropocentrism to ecocentrism. The initial connotation of ESD was to help students recognize the destructive effects of human activity on the environment and take action to improve the situation (Gokool-Ramdoo and Rumjaun, 2017). At the 2015 UNESCO World Conference on ESD, UNESCO stated that ESD can equip all people with the values, competencies, knowledge, skills, and Critical Thinking needed to adapt to the future (Gokool-Ramdoo and Rumjaun, 2017). This indicates that the core of ESD does not lie in simply adding knowledge. Rather, it lies in reshaping learners' values and agency for navigating uncertain futures (UNESCO, 2015, 2020). Accordingly, education itself must first undergo a paradigm shift.

This shift means that the mission of education is no longer confined to the transmission of knowledge. Instead, it increasingly emphasizes critical reflection, reflective learning, collaborative participation, and real-world action as means of promoting both individual and societal transformation. Sterling argued that if education has the capacity to transform society, then education itself must also be transformed (Huckle and Sterling, 2002). Specifically, education is both part of the problem and a vital pathway toward the solution. Consistent with this view, Jacobs and colleagues argued that in the era of sustainable development, education has moved beyond traditional knowledge dissemination toward the cultivation of critical thinking, innovation, and social participation (Jacobs, 2010). Thus, the real challenge of ESD is not what content to add, but whether course objectives, teaching methods, and evaluation mechanisms can be reconstructed to genuinely support the development of learners' sustainability-related capacities.

In recent years, international research on ESD has gradually shifted from value advocacy to the construction of competence frameworks. Wiek et al. (2011) proposed a set of core sustainability competencies, including systems thinking, anticipatory competence, normative competence, strategic competence, and interpersonal competence, which provided an important reference for curriculum reform in higher education. Brundiers et al. (2021) further advanced the integration and consensus-building of sustainability competence frameworks in higher education, while the GreenComp framework issued by the European Union systematically defined sustainability competencies across four dimensions: embodying sustainability values, embracing complexity, envisioning sustainable futures, and acting for sustainability (Bianchi et al., 2022). These studies show that ESD is moving from a macro-level concept toward an educational framework that is designable, implementable, and evaluable. Despite continued development of competence framework research, the construction of discipline-adapted evaluation models at the course level remains underdeveloped, especially in practice-oriented design disciplines, where consistency among philosophy, evaluation, and teaching is still difficult to achieve.

### 2.2 The transformation of design education: from aesthetic form-giving to ecological symbiosis

As an interdisciplinary field that connects science and technology, engineering, art, and social development, design disciplines are shifting from traditional aesthetic form-giving toward ecological symbiosis while increasingly emphasizing the integration of creativity, interdisciplinary capabilities, and sustainability literacy. Design is no longer understood merely as the optimization of form and function. Instead, it is increasingly regarded as an intervention in and reconstruction of complex systems, social relations, and future ways of living. Buchanan argued that design addresses “wicked problems,” which are characterized by high interdependence and uncertainty and cannot be solved through the logic of a single discipline (Buchanan, 1992). In the context of ESD, design practice has expanded from product improvement to system transformation (Ceschin and Gaziulusoy, 2016). Meyer and Norman further emphasized that 21st-century design education must move beyond traditional disciplinary divisions and isolated skill training to place greater emphasis on understanding complex systems, interdisciplinary collaboration, technological literacy, social responsibility, and the ability to respond to uncertain futures (Meyer and Norman, 2020). Annan-Diab and Molinari argued that interdisciplinary collaboration is a key pathway for implementing ESD (Annan-Diab and Molinari, 2017). Although researchers have begun to pay attention to reflective practice in the design process, most evaluation systems still rely heavily on the uniqueness and aesthetic quality of final products rather than on students' decision-making logic when responding to complex social and ecological constraints (Gero et al., 2023; Cash et al., 2023). For design education, this means that courses should not only encourage students to integrate perspectives from art, science, technology, and sociology, but also help them establish systemic connections among humans, nature, technology, and society in authentic problem contexts. Specifically, creativity in design education is no longer merely formal innovation, but a capacity for sustainable innovation oriented toward complex real-world problems.

However, this transformation is highly uneven across institutions. In China's regional public undergraduate universities, which constitute the majority of the national higher education system, design programs often originated in traditional fine arts education. As a result, teachers' personal experience has long served as the primary standard for learning evaluation. The resulting subjectivity and singularity impose clear limitations on traditional course evaluation systems. Meanwhile, conservative teacher-centered modes of instruction remain widespread, and Course Evaluation continues to focus strongly on outcomes and subjective experience (Zhu, 2015; Saris, 2020; Zhu et al., 2019). Although China's education modernization 2035 emphasizes the construction of an innovative talent cultivation system (Central Committee of the Communist Party of China, 2019), the lag in evaluation reform has prevented ESD principles from effectively being integrated into micro-level teaching practice. This contradiction, in which educational goals are transformed more quickly than evaluation systems, highlights the urgency of constructing an evaluation system for design education that fits the context of Chinese regional universities.

### 2.3 The evolution of evaluation paradigms: from outcome verification to assessment as learning

Against the background of mass higher education and the AI revolution, the educational era centered on degree attainment is giving way to an era of lifelong learning (Håkansson Lindqvist et al., 2024). Thus, both learning paradigms and evaluation paradigms in universities are undergoing profound change. With the growing influence of constructivist philosophy, the learner-centered perspective has become increasingly prominent. Learning is no longer understood as the passive reception of existing knowledge, but as an active process of meaning construction through inquiry, collaboration, reflection, and situated interaction. In design education, evaluation is shifting from the assessment of learning (AoL), which verifies outcomes, toward assessment as learning (AaL). In this evaluation, itself becomes a teaching mechanism that promotes reflection and stimulates creative decision-making. The meaning, form, and agents of evaluation have all changed substantially.

John Biggs' theory of constructive alignment, proposed in *Teaching for Quality Learning at University*, has been further extended in AI-enabled educational settings. Recent studies have emphasized the dynamic alignment of objectives, teaching, and evaluation and suggested that technological tools should be used to achieve personalized alignment (Biggs et al., 2022). Boud and Falchikov argued that evaluation should serve long-term learning capacity, a view supported by more recent studies, especially through the development of evaluative judgment. In interdisciplinary design education contexts, evaluation needs to support both skill acquisition and lifelong learning literacy (Tai et al., 2022; Tai et al., 2023). The framework of evaluative judgment proposed by Carless et al. has been empirically validated as central to the cultivation of creative talent. Through iterative feedback cycles, students can autonomously adjust the direction of their design practice and improve the quality of innovative decision-making (Carless and Boud, 2021; Carless and Boud, 2018). More recent interdisciplinary studies have further suggested that evaluative judgment in design education should be integrated with sustainability thinking to form a closed loop of evaluation, reflection, innovation, and sustainability (Burns, 2024).

These developments indicate that the position and dimensions of course evaluation essentially reflect the educational values embedded in a curriculum and embody value judgments about learning activities. This is especially true in weak-paradigm fields such as design disciplines, where evaluation has a strong steering effect. For ESD-oriented creativity courses in design disciplines, if evaluation remains limited to summative scores, single-teacher judgment, and final displays of outcomes, it cannot effectively identify students' development in critical thinking, interdisciplinary integration, collaborative learning, and responsibility awareness. In the Chinese context of design education in particular, traditional course evaluation often takes the form of a simple weighting of attendance and final project work, resulting in a static, instrumental approach that cannot respond to the dynamic development of student creativity. This suggests that the genuine transformation of evaluation in design education is not merely a change in evaluative form, but a reconstruction of curricular values and conceptions of learning.

### 2.4 The evaluation dilemma of design creativity and its integration with ESD

Creativity is the driving force behind design innovation and a core literacy in design education (Li et al., 2024). Creativity is the external manifestation of creative thinking (Xu et al., 2022); therefore, the development and evaluation of creativity are also, in essence, the development and evaluation of the process through which creative thinking emerges. Although many studies have noted that creativity lacks a precise definition, it is widely accepted that creativity includes at least two common attributes: novelty and appropriateness (Abraham, 2022). In design disciplines, creativity must also take into account imagination, functional relevance, contextual suitability, and technical feasibility (Raghunath et al., 2023). As design increasingly intersects with other disciplines, design creativity is becoming more interdisciplinary, systemic, and situated (Zhang, 2023), rather than being understood simply as individual inspiration or formal novelty.

Within the context of ESD, however, design creativity must additionally incorporate ecological ethics, social responsibility, and technical feasibility (Fan, 2025). Although research on evaluating design creativity is abundant, several clear limitations remain. First, evaluation indicators often center on technical proficiency, project completion, or expressive creativity, while deeper dimensions such as critical thinking, interdisciplinary literacy, and social responsibility are less systematically addressed. Second, evaluation methods still rely heavily on teachers' personal judgment, leading to substantial subjectivity and considerable variation in scoring among evaluators. Third, many evaluations remain more focused on outcomes than on students' creativity development during exploration, trial and error, reflection, and iteration. For ESD-oriented design education, this evaluation logic, which emphasizes skills over thinking and outcomes over process, is clearly inadequate for fostering Sustainable Creativity.

International research trends increasingly emphasize the role of authentic assessment, formative assessment, and performance assessment in creativity development in design education. Through project processes, public displays of work, peer review, portfolios, and continuous feedback, these approaches provide a more comprehensive understanding of students' creative performance in complex tasks (Yusoff, 2025). Such studies offer an important insight for evaluating design creativity: creativity is not a result to be measured once at the end of a course, but something gradually constructed through course philosophy, content design, teaching strategies, interaction and feedback, and evaluation mechanisms (Penaluna and Penaluna, 2009). For this reason, if ESD is to be deeply integrated with creativity development, course evaluation cannot be treated merely as a tool for judging outcomes. It must instead occupy a central position in curriculum reform.

Nevertheless, existing studies remain insufficient in integrating creativity evaluation in design education with ESD. On the one hand, ESD research tends to emphasize interdisciplinarity, systems thinking, and action competence, but seldom translates these ideas into mechanisms for evaluating creativity in design courses. On the other hand, research on creativity in design education focuses on innovative thinking and design performance but rarely incorporates sustainability values, ecological ethics, and social responsibility systematically into evaluation frameworks. Specifically, research on how creativity should be evaluated has not yet genuinely converged with research on how sustainability should be integrated into the curriculum. This gap constitutes a key point of entry for the present study.

### 2.5 Research gaps and the exploratory positioning of this study

In summary, although theoretical discussions of ESD, design education, creativity development, and course evaluation have become increasingly rich, integrated research remains insufficient. First, the evaluation of design creativity has not yet been deeply integrated with ESD, and the definition and measurement of Sustainable Creativity remain underdeveloped. Second, within design disciplines, ESD-oriented course evaluation still lacks a systematic tool that integrates qualitative meaning with quantitative analysis, especially in course contexts characterized by multidimensionality, fuzziness, and subjectivity. Third, traditional course evaluation paradigms still largely follow the linear logic of “objectives-content-process-evaluation” and rarely work backward from course philosophy and evaluation mechanisms to drive the reconstruction of teaching and content. Consequently, they are poorly suited to the demands of interdisciplinary and inquiry-based teaching. In response, this study attempted to construct a course evaluation system for creativity courses in design disciplines oriented to ESD, drawing on four dimensions—course philosophy, course content, course teaching, and course evaluation—and integrating AHP-EWM with FCE to address the practical challenges of multidimensional indicators, complex weighting structures, and fuzzy judgments in the evaluation of creativity courses.

Notably, the empirical part of this study is exploratory. Its purpose is not to derive universal conclusions directly but to validate the logical feasibility and preliminary applicability of the model through the case of “H” University, a regional university in China with a certain degree of representativeness. Accordingly, the study is more concerned with whether the model can identify the key contradictions and leverage points in course transformation. Its broader applicability across regions, institutional types, and disciplinary orientations still requires further testing in more diverse contexts.

## 3 Materials and methods

**Figure 2:**
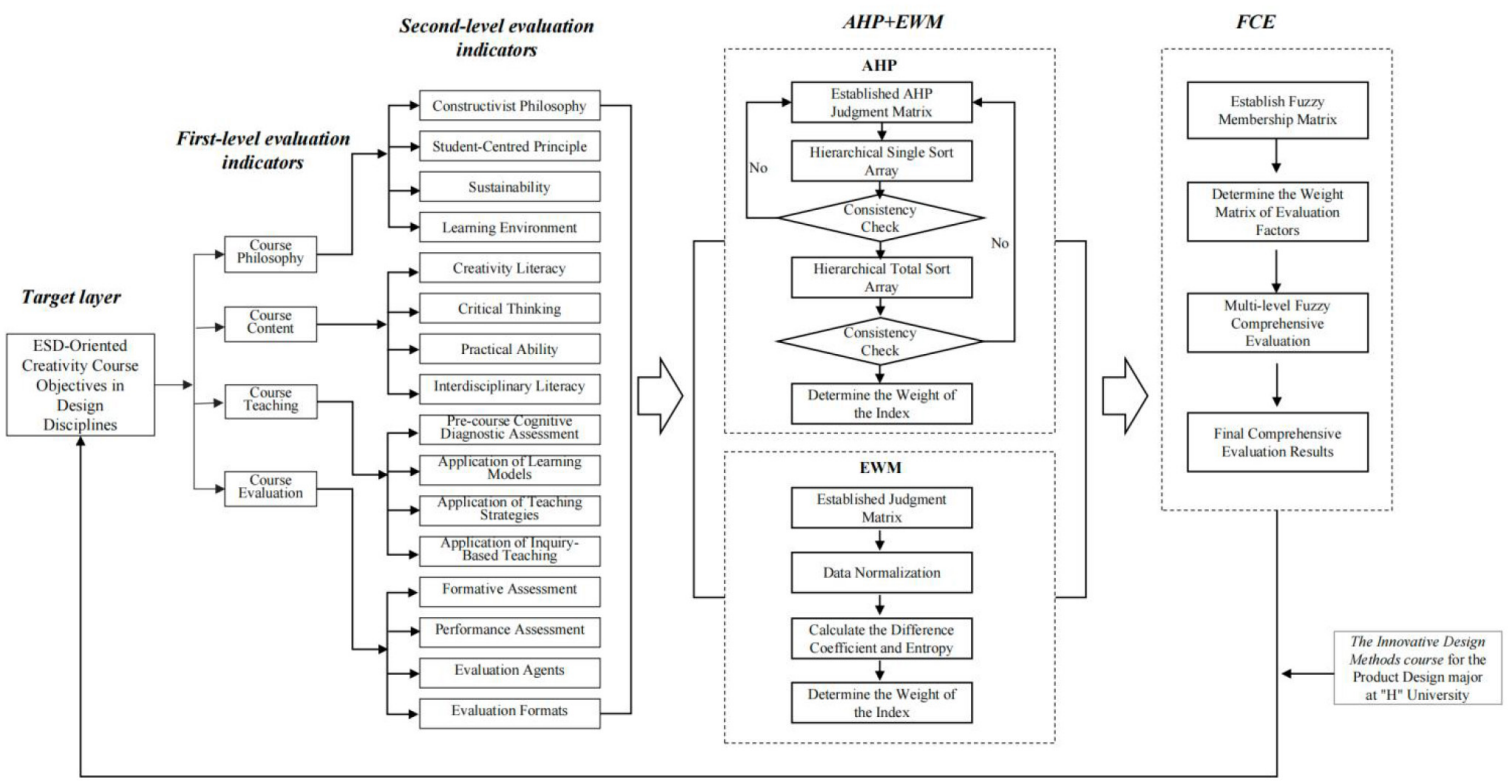
Computational procedure of the evaluation model and framework of the AHP-EWM-FCE method.

Course evaluation is the process of making value judgments about educational activities, processes, and outcomes within a curriculum and can serve as a reference standard for future educational practice (Leathwood and Phillips, 2000). Such evaluation is not only used to measure the quality and effectiveness of educational processes, but also serves a guiding function throughout teaching and learning. Course evaluation involves multiple aspects, including course philosophy, course content, and course implementation. It therefore has the characteristics of multiple attributes, multiple levels, and fuzziness, which make quantitative analysis difficult. This study adopted a three-stage research procedure of indicator construction, combined weighting (AHP-EWM), and fuzzy comprehensive evaluation, as illustrated in Figure 2. By comprehensively applying AHP and EWM, the weights of specific indicators under different dimensions were calculated in an integrated manner. This approach made it possible to derive subjective weight data through AHP while reducing subjective influence through EWM, thereby enabling a more intuitive comparison of indicator importance. In this way, subjective and objective perspectives, as well as quantitative and qualitative research, were integrated to construct a scientific and rational course evaluation system for creativity courses in design disciplines. This system was then combined with FCE and applied to the course Innovative Design Methods in the Product Design program at “H” University, a regional university in China.

There is already substantial research on the application of combined weighting (AHP-EWM) in decision-making and on the use of FCE in course evaluation. For example, Xia et al. (2024) and Liu et al. (2022) adopted AHP to construct an online course quality evaluation system. Then, they combined it with FCE to quantify the quality of online courses (Xia et al., 2024; Liu et al., 2022). Li (2023) also evaluated the effectiveness of general education courses in vocational colleges using an AHP-based FCE approach. He (2022) constructed an evaluation model for blended foreign-language teaching based on AHP. Chen (2023) selected fuzzy AHP, which integrates fuzzy mathematics and AHP, to evaluate teaching quality.

### 3.1 Indicator construction

**Figure 3:**
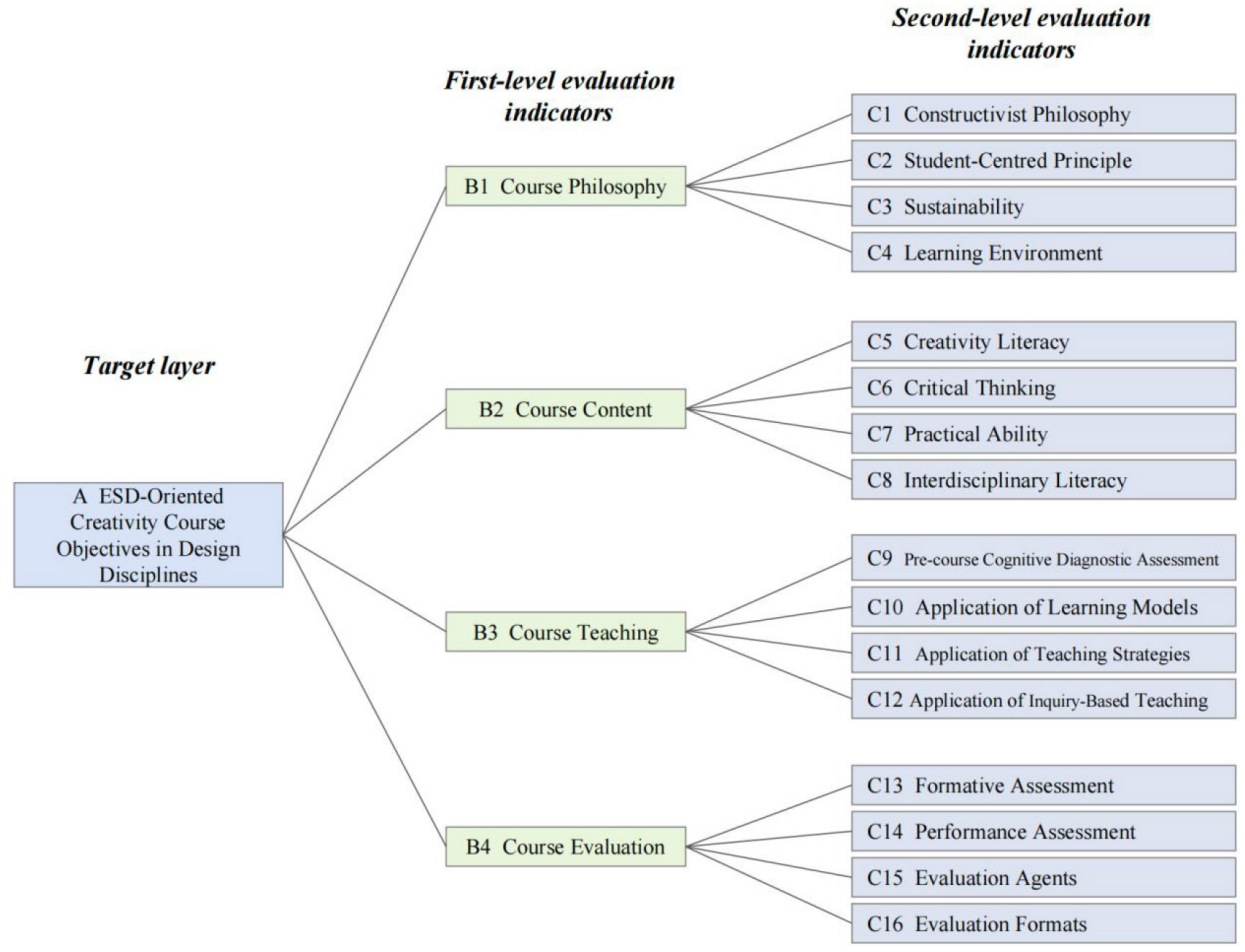
Hierarchical evaluation model for creativity courses in design disciplines oriented to ESD. The hierarchical model constructed in this study integrates ESD goals with the core objective of creativity development. The model contains three layers: the Target Layer (A), which governs the overall system; the Criteria Layer (B1–B4), which establishes four pillars—course philosophy, course content, course teaching, and course evaluation—and reflects the entire process from value orientation to implementation and feedback; and the Alternative Layer (C1–C16), which refines these into 16 observable and measurable indicators.

Through literature analysis, ESD policy review, and expert consultation, this study adopted a constructivist view of learning, treated creativity in design disciplines as the core literacy, and applied ESD-oriented principles to construct sustainable, extensible, and interdisciplinary course objectives. By comparing the strengths and weaknesses of existing evaluation indicator systems and repeatedly consulting experts and scholars in design disciplines, the study identified a target layer representing the overall objective of course evaluation, denoted as A. Based on this overall objective, four dimensions—course philosophy, course content, course teaching, and course evaluation—were established as first-level indicators, denoted as B. Each first-level indicator included four second-level indicators, denoted as C. In this way, a multi-level course evaluation indicator system for creativity courses in design disciplines was ultimately constructed (see Figure 3).

Among the four first-level indicators, course philosophy refers to the methodological approach and basic stance for implementing course objectives. It reflects the fundamental viewpoint of the course and provides the ideological foundation for ensuring the attainment of course objectives. Course content specifies the main framework and content set through which the objectives are realized. Course teaching refers to the alignment between teaching methods and target attainment, essentially representing the unity of method and objective. Course evaluation is a highly important technical means and an effective application of methodology. Following the principle of aligning evaluation with objectives, 16 second-level indicators were derived to correspond to specific content, as shown in Table 1.

**Table 1:** Connotations of the evaluation indicators.

Target layer	Criteria layer	Alternative layer	Indicator connotation
A ESD-oriented creativity course objectives in design disciplines	B1 Course philosophy	C1 Constructivist philosophy	Knowledge is actively constructed through experience. Students are expected to construct knowledge through information gathering, while teachers support them in acquiring knowledge through inquiry and critical thinking based on prior experience. In creativity courses, students should integrate sustainability principles into design projects and develop creative solutions through autonomous exploration and continuous revision.
		C2 Student-centered principle	Learning is reconstructed as a process of co-constructing meaning. Its essence lies in shifting learning from knowledge transmission to meaning construction. Through dynamic interaction with teachers and peers, students not only acquire knowledge but also develop the core literacy of learning, creating, and reflecting throughout design practice, while strengthening their sense of responsibility and improving design thinking through collaboration and feedback.
		C3 Sustainability	This indicator emphasizes the integration of values, knowledge, and action. It aims to develop students' capacity to survive, adapt, and innovate in a complex world by integrating values, knowledge, and skills. This capacity is directed not only toward responding to environmental crises but also toward enabling students to apply systems thinking to design problems in ways that account for social equity, economic transformation, and ecological balance.
		C4 Learning environment	This indicator concerns the integrated presentation of material, social, and psychological environments. It embodies educational philosophy across these dimensions. The environment of design courses should go beyond a simple physical space to provide work settings and team atmospheres that support active inquiry and continuously stimulate creativity through an adaptive and inclusive educational ecology during students' cognitive development.
	B2 Course content	C5 Creativity literacy	This indicator integrates imagination, novelty, and appropriateness. It focuses on the application of innovative design methods and combines imagination, novelty, and functionality. Evaluation should not only address whether a design solution can be implemented, but also students' systematic integrative ability from problem discovery to problem solving, as well as their originality and optimization ability during solution iteration.
		C6 Critical thinking	This indicator is intended to help students move from task completion to reflective creation in design projects. In design expression, students are guided to develop habits of accepting questioning and reasoning critically so that they can examine the ethical and social responsibilities behind design decisions, ensure that design solutions conform to sustainability principles, and enhance rational judgment.
		C7 Practical ability	The essence of practical ability lies in transforming theoretical knowledge into the literacy required to solve real-world problems. Through project-based practice, students can develop self-efficacy when facing design challenges. This ability is reflected in learners' capacity to maintain rational analysis in complex practice while improving creative problem solving through continuous trial and revision.
		C8 Interdisciplinary literacy	This indicator seeks to break down disciplinary boundaries and help students establish a cognitive framework that recognizes the interconnectedness of the world. It guides students to integrate perspectives from art, science, and sociology, stimulates exploratory interest in the process of integrating information from different fields, and promotes innovative design solutions capable of responding to complex contexts
	B3 Course teaching	C9 Pre-course cognitive diagnostic assessment	This indicator functions as a mechanism for calibrating the starting point of teaching. By diagnosing students' pre-course status, teaching objectives can align with both standards and with students' actual needs. This helps improve teaching efficiency, enables students to participate actively in course planning under clear objective guidance, and supports a shift from passive reception to active learning.
		C10 Application of learning models	This indicator emphasizes the integration of deep learning, strategic learning, and surface learning. Students are guided to switch learning strategies accurately for different design tasks, thereby avoiding both mechanical imitation and inefficient blind inquiry, and gradually developing effective learning methods as the course progresses.
		C11 Application of teaching strategies	This indicator reflects teachers' dynamic adjustments to grouping and differentiation based on students' learning progress, ensuring that teaching strategies meet individual learning needs. Such strategies help create a learning environment that encourages interaction and collaboration while cultivating teamwork and social skills alongside cognitive development.
		C12 Application of inquiry-based teaching	This indicator emphasizes the creation of authentic problem situations and the construction of a learning community that involves teacher-student and student-student interactions. In the interactive processes of information collection, analytical reasoning, and practical verification, learners construct knowledge autonomously and develop core literacy in critical thinking, problem-solving, and collaborative innovation.
	B4 Course evaluation	C13 Formative assessment	Formative assessment makes feedback and improvement its core mechanism. Through ongoing process-oriented assessment, it dynamically monitors students' learning progress, clarifies the level they are expected to achieve, and feeds evaluation results back into teaching in a timely manner, thereby promoting continuous optimization of course content and learning methods and supporting the achievement of teaching objectives.
		C14 Performance assessment	Performance assessment transforms evaluation into a constructive practice within the learning community by asking students to present, demonstrate, or exhibit learning outcomes. It evaluates not only results but also the reflective thinking shown during presentation, and thus serves as an important tool for promoting sustainable learning and laying the foundation for lifelong learning.
		C15 Evaluation agents	The essence of this indicator is to break the teacher-centered monopoly on evaluation and construct an evaluation ecology involving teachers, students, peers, and industry experts. This helps students develop a sense of quality and supports a shift from passively receiving evaluation to actively managing growth, thereby improving the quality of design education in multiple dimensions.
		C16 Evaluation formats	This indicator transforms evaluation from a judgment tool into an empowering tool. When students move from passively receiving evaluation to actively participating in its design, implementation, and reflection, evaluation itself becomes an important medium for cognitive construction and collaborative learning, thereby fulfilling the educational purpose of promoting student development.

The table presents clear operational definitions for the 16 second-level indicators. These definitions ensure that the indicators are theoretically grounded and can accurately guide observation and measurement in practice, thereby establishing a shared basis for subsequent expert scoring and weight calculation.

### 3.2 Weight calculation of the evaluation indicators

#### 3.2.1 AHP weighting

After determining the indicators in the ESD-oriented evaluation system for creativity courses in design disciplines, it was necessary to compare the relative importance of each indicator and calculate the corresponding weights. This study adopted the AHP proposed by Thomas L. Saaty. AHP is a group decision-making method that decomposes a complex problem into several decision-related indicators (Saaty, 2003). Based on the relationships among these indicators, it organizes them into ordered hierarchical levels such as the target layer, criteria layer, and alternative layer. Indicators at each level have their own position, and there are interconnections across levels and among indicators. A hierarchical model is then established based on these relationships, followed by qualitative and quantitative analysis to support decision-making. The specific steps were as follows: (1) establishing the hierarchical structure model; (2) constructing the judgment matrix and conducting pairwise comparisons of indicators using the 1–9 scale; (3) performing single-level ranking to determine the weights of indicators within each level; (4) conducting the consistency test of judgment matrix; and (5) performing overall ranking to determine the global weights of indicators.

(1) Establishment of the hierarchical structure model

Based on ESD principles, this study constructed a hierarchical evaluation model for creativity courses in design disciplines (Table 2). The model consisted of three levels: the target layer (A), the criteria layer (B1–B4), and the alternative layer (C1–C16). The target layer specified that the course aimed to develop students' creativity and sustainability-related abilities. The criteria layer included four core dimensions: course philosophy, course content, course teaching, and course evaluation. The alternative layer refined these dimensions into 16 specific indicators covering the key elements of course implementation and evaluation. The model had a clear structure and distinct hierarchy, which facilitated the scientific, systematic evaluation of creativity courses in design disciplines and effectively supported the continuous improvement of course quality.

**Table 2:** Structure of the constructed evaluation model.

Target level (A)	First-level evaluation indicators (B)	Second-level evaluation indicators (C)
A ESD-oriented creativity course objectives in design disciplines	B1 Course philosophy	C1 Constructivist philosophy
		C2 Student-centered principle
		C3 Sustainability
		C4 Learning environment
	B2 Course content	C5 Creativity literacy
		C6 Critical thinking
		C7 Practical ability
		C8 Interdisciplinary literacy
	B3 Course teaching	C9 Pre-course cognitive diagnostic assessment
		C10 Application of learning models
		C11 Application of teaching strategies
		C12 Application of inquiry-based teaching
	B4 Course evaluation	C13 Formative assessment
		C14 Performance assessment
		C15 Evaluation agents
		C16 Evaluation formats

(2) Constructing the judgment matrix

$$A=\left[\begin{array}{ccccc} a_{11} & a_{12} & \cdots & \cdots & a_{1n}\\ a_{21} & a_{22} & \cdots & \cdots & a_{2n}\\ \cdots & \cdots & a_{ij} & \cdots & \cdots \\ \cdots & \cdots & \cdots & \cdots & \cdots \\ a_{n1} & a_{n2} & \cdots & \cdots & a_{nn} \end{array}\right]$$

In the matrix, *a*_*ij*_ denotes the relative importance between *A*_*i*_ and *A*_*j*_. If the former is more important, then *a*_*ij*_ > 1; if both are equally important, then *a*_*ij*_ = 1. The standards for relative importance are as shown in Table 3:

**Table 3:** Standards for relative importance.

Scale	Meaning description
1	Two factors are equally important
3	The former factor is slightly more important
5	The former factor is obviously more important
7	The former factor is strongly more important
9	The former factor is extremely more important
2, 4, 6, 8	Intermediate values between two adjacent judgments
Reciprocal	Reverse comparison equals the reciprocal of the above

(3) Calculating the weight vector of indicators

1) Normalize the matrix using the following formula (Equation 1):

$$\begin{array}{l} b_{ij}=\frac{a_{ij}}{\overset{n}{\underset{i=1}{\sum }}a_{ij}}\;\left(i,j=1,2,\cdots n\right) \end{array}$$ (1)

Where *a*_*ij*_ is the value in the *i*-th row and *j*-th column of the judgment matrix *A*; *b*_*ij*_ is the corresponding value in the normalized matrix.

2) Sum the elements of each row in the matrix:

$$\begin{array}{l} \bar{w_{i}}=\overset{n}{\underset{j=1}{\sum }}b_{ij}\;\left(i,j=1,2,\cdots n\right) \end{array}$$ (2)

3) Normalize the above results (Equation 3):

$$\begin{array}{l} w_{i}=\frac{\bar{w_{i}}}{\overset{n}{\underset{i=1}{\sum }}\bar{w_{i}}}\;\left(i=1,2,\cdots n\right) \end{array}$$ (3)

where *w*_*i*_ is the weight of the *i*-th indicator.

4) Calculate the maximum eigenvalue of the judgment matrix *A* (Equation 4):

$$\begin{array}{l} \lambda_{\max}=\frac{1}{n}\overset{n}{\underset{i=1}{\sum }}\frac{{\left(Aw\right)}_{i}}{w_{i}} \end{array}$$ (4)

Where *n* is the order of the matrix; *A* is the judgment matrix; *w*_*i*_ is the weight of the *i-*th indicator; λ_max_ is the maximum eigenvalue of matrix *A*.

5) Consistency check: for the vector and eigenvalues obtained above, perform a consistency test. If the test is passed, the judgment matrix is considered reasonable and meaningful. The consistency index CR (see Table 4) is calculated as follows (Equation 5):

$$\begin{array}{l} CI=\frac{\lambda_{\max}-n}{n-1} \end{array}$$ (5)

For the value of *n*, refer to the table above to obtain the RI value. The consistency ratio CR is then calculated as (Equation 6):


$$\begin{array}{l} \text{CR}=\frac{\text{CI}}{\text{RI}} \end{array}$$ (6)

When *CR*<*0.1*, the consistency test is satisfied, and the weight results are considered valid.

**Table 4:** Random consistency index (RI).

Primary indicator	1	2	3	4	5	6	7	8
RI	0	0	0.58	0.9	1.12	1.24	1.32	1.41

#### 3.2.2 Judgment matrix construction and weight calculation

Constructing the judgment matrix was the key step in this study and formed the foundation for weight calculation. To ensure scientific rigor and objectivity, this study invited 16 experts engaged in teaching and research in design disciplines as consultees. The experts included front-line teachers and teaching administrators from regional undergraduate institutions in China. In terms of demographic characteristics, there were nine men and seven women, aged 29–54 years, with a mean age of 43.25 years. Six experts held doctoral degrees (37.5%), and 10 held master's degrees (62.5%). Their professional ranks included four professors, seven associate professors, three lecturers, and two teaching assistants, all with disciplinary backgrounds in design disciplines (see Table 5). All experts had substantial teaching experience in creativity courses and a strong theoretical understanding of disciplinary development and ESD principles. Given the complexity of evaluation in design education, the judgment matrix was not determined by a single researcher. Instead, this study adopted a consensus-oriented decision path within Group AHP (Ishizaka and Nemery, 2013; Akaa et al., 2016) and followed a rigorous process of independent scoring, group discussion, and aggregation into a consensus matrix to avoid bias arising from individual subjective judgment (Saaty, 2008).

**Table 5:** Demographic information of the experts.

No.	Item	Category/value	Number/value	Proportion (%)
1	Total experts	–	16	100
2	Gender	Men	9	56.25
		Women	7	43.75
3	Age	Age range	29–54 years	–
		Average age	43.25 years	–
		Median age	43.5 years	–
4	Degree	Master's	10	62.5
		Doctorate	6	37.5
5	Title	Professor	4	25
		Associate professor	7	43.75
		Lecturer	3	18.75
		Assistant lecturer	2	12.5
6	Specialization	Product design	5	31.25
		Visual communication design	4	25.00
		Environmental design	4	25.00
		Other	3	18.75

To make full use of collective expertise, the following procedure was implemented. First, the research team introduced the study topic and the connotations of the evaluation indicators to each expert in detail. It provided video explanations to ensure a shared understanding of ESD principles. In the first round, the experts independently assigned scores using the 1–9 scale. Subsequently, for indicators with notable disagreement, the research team organized focused discussions among the experts to facilitate an in-depth review of the evaluation dimensions through mutual exchange. A consensus-based Judgment Matrix was then formed through aggregation. This consensus-building process based on group decision-making can effectively address the complexity and subjectivity commonly found in evaluation in design education (Osgood and Johnston, 2022).

Finally, based on the consensus matrix, AHP was used for calculation, and the consistency test of judgment matrix was conducted rigorously. The results showed that the CR values of all matrices were below 0.1, meeting the required scientific standard. The final indicator weights and related results are presented in Table 6 (see Supplementary Table S1 for the complete set of secondary judgment matrices).

**Table 6:** Judgment matrix for the criteria layer.

Criteria layer	B1 Course philosophy	B2 Course content	B3 Course teaching	B4 Course evaluation
B1 Course philosophy	1	4	3	2
B2 Course content	1/4	1	1/2	1/3
B3 Course teaching	1/3	2	1	1/2
B4 Course evaluation	1/2	3	2	1

In matrix form, denoted by A, it is as follows:


$$\begin{array}{c} A=\left[\begin{array}{cccc} 1 & 4 & 3 & 2\\ 1/4 & 1 & 1/2 & 1/3\\ 1/3 & 2 & 1 & 1/2\\ 1/2 & 3 & 2 & 1 \end{array}\right] \end{array}$$

Normalizing the column vectors of judgment matrix A, we obtain matrix B:


$$\begin{array}{c} B=\left[\begin{array}{cccc} 0.4800 & 0.4000 & 0.4615 & 0.5217\\ 0.1200 & 0.1000 & 0.0769 & 0.0870\\ 0.1600 & 0.2000 & 0.1538 & 0.1304\\ 0.2400 & 0.3000 & 0.3077 & 0.2609 \end{array}\right] \end{array}$$

Summing the elements of each row vector of matrix B according to Equation 2:


$$\begin{array}{c} \bar{W}=\left[\begin{array}{c} 1.8633\\ 0.3839\\ 0.6443\\ 1.1086 \end{array}\right] \end{array}$$

Normalizing the vector $\bar{W}$ to obtain the weight *W*:


$$\begin{array}{c} W=\left[\begin{array}{c} 0.4658\\ 0.0960\\ 0.1611\\ 0.2771 \end{array}\right]\\ AW=\left[\begin{array}{cccc} 1 & 4 & 3 & 2\\ 1/4 & 1 & 1/2 & 1/3\\ 1/3 & 2 & 1 & 1/2\\ 1/2 & 3 & 2 & 1 \end{array}\right]\left[\begin{array}{c} 0.4658\\ 0.0960\\ 0.1611\\ 0.2771 \end{array}\right]=\left[\begin{array}{c} 1.8872\\ 0.3853\\ 0.6469\\ 1.1201 \end{array}\right] \end{array}$$

Calculate the maximum eigenvalue of the judgment matrix *A*, and the result is as follows:


$$\begin{array}{c} \lambda_{\max}=\frac{1}{4}\left(\frac{1.8872}{0.4658}\text{+}\frac{0.3853}{0.0960}\text{+}\frac{0.6469}{0.1611}\text{+}\frac{1.1201}{0.2771}\right)=\text{4.0310} \end{array}$$

Subsequently, a consistency check is performed by calculating the consistency index CI:


$$\begin{array}{c} CI=\frac{\lambda_{\max}-n}{n-1}=\frac{\text{4.0310}-4}{4-1}=\text{0.0103} \end{array}$$

By consulting Table 6 for the average random consistency index (RI), we computed the random consistency ratio as:


$$\begin{array}{c} CR=\frac{CI}{RI}=\frac{\text{0.0103}}{0.\text{9}}=\text{0.0115}<0.10 \end{array}$$

Since CR < 0.1, the construction of the judgment matrix is deemed reasonable, and thus the indicator weights are calculated as shown in the table below (see Table 7).

**Table 7:** Criteria layer indicator weight calculation results.

Criteria layer	Weight
B1 Course philosophy	0.4658
B2 Course content	0.096
B3 Course teaching	0.1611
B4 Course evaluation	0.2771

Following the same procedure, the weights of the alternative layer indicators were further calculated, and all matrices passed the consistency test. The results are shown in Table 8.

**Table 8:** Criteria layer and alternative layer weight calculation results.

Criteria layer	Weight	Alternative layer	Weight	Global weight
B1 Course philosophy	0.4658	C1 Constructivist philosophy	0.284	0.1323
		C2 Student-centered principle	0.1715	0.0799
		C3 Sustainability	0.4709	0.2193
		C4 Learning environment	0.0736	0.0343
B2 Course content	0.096	C5 Creativity literacy	0.4839	0.0465
		C6 Critical thinking	0.168	0.0161
		C7 Practical ability	0.0685	0.0066
		C8 Interdisciplinary literacy	0.2795	0.0268
B3 Course teaching	0.1611	C9 Pre-course cognitive diagnostic assessment	0.1237	0.0199
		C10 Application of learning models	0.0731	0.0118
		C11 Application of teaching strategies	0.3001	0.0483
		C12 Application of inquiry-based teaching	0.503	0.081
B4 Course evaluation	0.2771	C13 Formative assessment	0.4959	0.1374
		C14 Performance assessment	0.2887	0.08
		C15 Evaluation agents	0.0858	0.0238
		C16 Evaluation formats	0.1296	0.0359

To maintain conciseness, this study details the judgment matrix for the criteria layer (Table 6). All matrices for the alternative layer were constructed following the identical consensus-building protocol and successfully passed the consistency test (all CR < 0.1). The complete set of matrices is available as Supplementary material or upon reasonable request (Supplementary Table S1).

**Figure 4:**
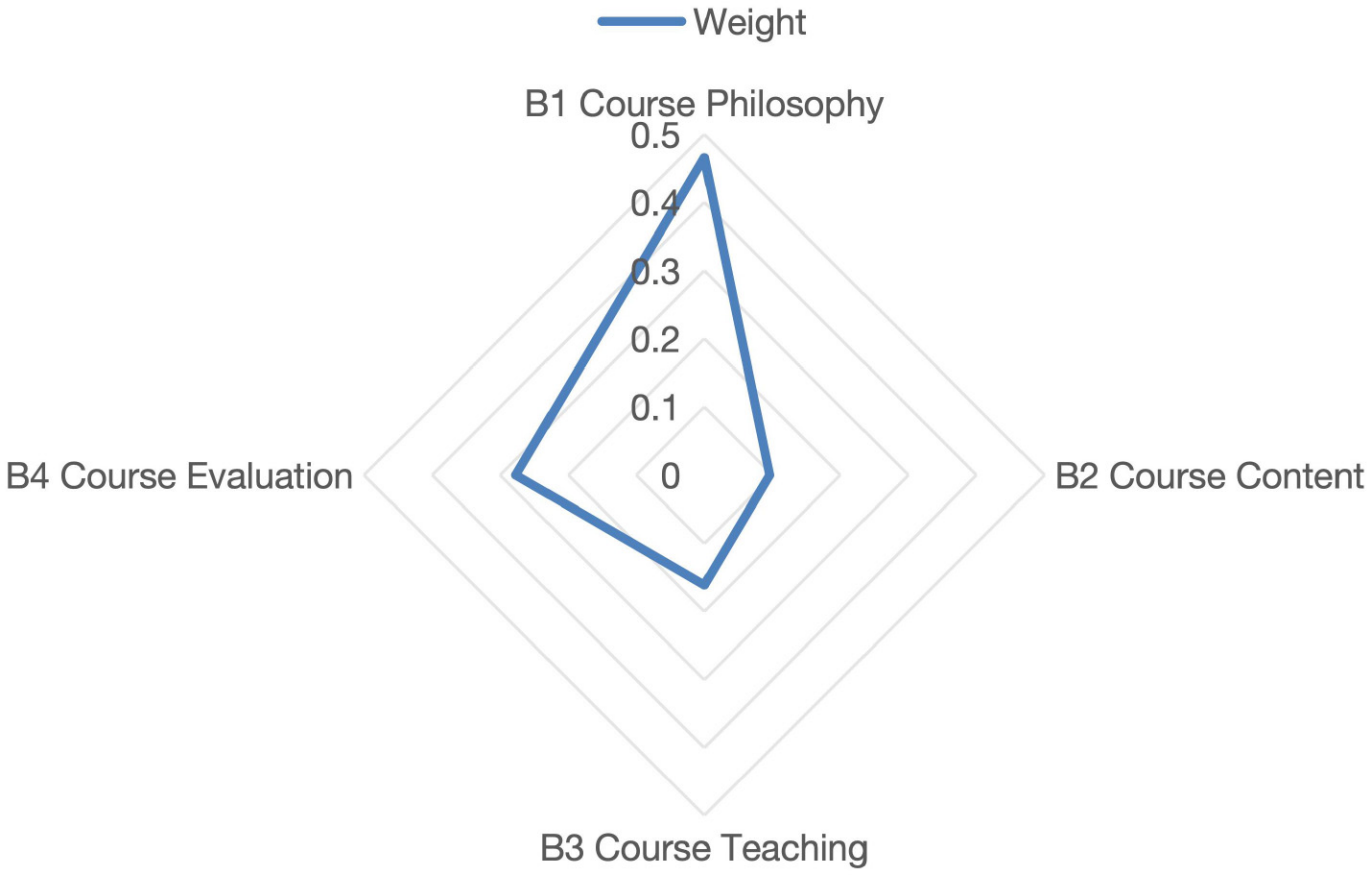
Radar chart of the Criteria Layer weights. The radar chart visually presents the relative magnitudes of the weights of the criteria layer indicators (B1–B4).

After aggregation, the radar chart of the criteria layer weights (Figure 4) is as follows:

#### 3.2.3 Weight revision using the entropy weight method

To compensate for the limitations of AHP, EWM was used to revise further the indicator weights obtained above. By combining the weights derived from AHP and EWM, this study not only reduced the high subjectivity associated with qualitative analysis but also determined the relative weights of the evaluation indicators more objectively, thereby making the weighting results more systematic and feasible. The specific steps are as follows:

(1) Before calculating the weights of each indicator, standardization processing is required to obtain standardized indicator values (Equation 7):

$$\begin{array}{cc} x_{ij}^{0} & \left(i=1,2,...,m,j=1,2,...,p\right): \end{array}$$

$$\begin{array}{c} x_{ij}^{0}=\frac{x_{ij}}{\max x_{j}} \end{array}$$ (7)

(2) After standardization, some indicator values may be very small or negative. For consistency and convenience of calculation, the standardized values are shifted to eliminate such situations (Equation 8):

$$\begin{array}{c} x_{ij}^{\prime }\text{=}H+x_{ij}^{0} \end{array}$$ (8)

Where H is the amplitude of the indicator shift, typically set to 1.

(3) Normalize the data using the proportion method. The formula is as follows (Equation 9):

$$y_{ij}=\frac{x_{ij}^{'}}{\sum_{i=1}^{n}x_{ij}^{'}}$$ (9)

(4) Calculate the entropy value of the *j*th indicator *e*_*j*_(*j* = 1, 2, ⋯⋯*p*) (Equation 10):

$$\begin{array}{c} e_{j}=-\frac{1}{\ln\;n}\overset{n}{\underset{i=1}{\sum }}y_{ij}\ln\;y_{ij} \end{array}$$ (10)

(5) Calculate the coefficient of variation for the *j*th indicator *g*_*j*_(*j* = 1, 2, …*p*) (Equation 11):

$$\begin{array}{c} g_{j}=1-e_{j} \end{array}$$ (11)

(6) Calculate the weight of the *j*th indicator using EWM (*j* = 1, 2, …*p*) (Equation 12):

$$\begin{array}{c} \omega_{j}=\frac{g_{j}}{\overset{p}{\underset{j=1}{\sum }}g_{j}} \end{array}$$ (12)

(7) Calculate the combined weight λ_*j*_(*j* = 1, 2, …*p*) (Equation 13):

$$\begin{array}{c} \lambda_{j}=\frac{w_{j}v_{j}}{\overset{n}{\underset{j=1}{\sum }}w_{j}v_{j}} \end{array}$$ (13)

Where *w*_*j*_ is the weight from the Entropy Weight Method; *v*_*j*_ is the weight from the AHP.

As demonstrated in the preceding indicator construction process, the evaluation indicators selected for course objectives are entirely qualitative. To address these qualitative indicators, data were acquired through the designed expert opinion scoring scales. These data were subsequently standardized according to the formulas provided in the text. By integrating the previously described calculation methods for entropy and entropy weights, the entropy values and weights for each indicator were determined as shown in Table 9.

**Table 9:** Indicator weights after revision using EWM.

Criteria layer	AHP weight	Entropy value	Difference coefficient	EWM weight	Combined weight	Alternative layer	AHP weight	Entropy value	Difference coefficient	EWM weight	Combined weight
B1 Course philosophy	0.4658	0.9892	0.0108	0.1938	0.3801	C1 Constructivist philosophy	0.2840	0.9819	0.0181	0.2607	0.3182
						C2 Student-centered principle	0.1715	0.9784	0.0216	0.3103	0.2287
						C3 Sustainability	0.4709	0.9871	0.0129	0.1859	0.3762
						C4 Learning environment	0.0736	0.9831	0.0169	0.2431	0.0769
B2 Course content	0.096	0.987	0.0126	0.2275	0.0919	C5 Creativity literacy	0.4839	0.8702	0.1298	0.1827	0.3866
						C6 Critical thinking	0.168	0.7632	0.2368	0.3335	0.2450
						C7 Practical ability	0.0685	0.8284	0.1716	0.2416	0.0724
						C8 Interdisciplinary literacy	0.2795	0.828	0.172	0.2422	0.296
B3 Course teaching	0.1611	0.9833	0.0167	0.301	0.2041	C9 Pre-course cognitive diagnostic assessment	0.1237	0.7949	0.2051	0.3147	0.1683
						C10 Application of learning models	0.0731	0.8491	0.1509	0.2315	0.0732
						C11 Application of teaching strategies	0.3001	0.8301	0.1699	0.2607	0.3384
						C12 Application of inquiry-based teaching	0.5030	0.8741	0.1259	0.1931	0.4201
B4 Course evaluation	0.2771	0.9846	0.0154	0.2777	0.3239	C13 Formative assessment	0.4959	0.8959	0.1041	0.1834	0.3996
						C14 Performance assessment	0.2887	0.8579	0.1421	0.2503	0.3175
						C15 Evaluation agents	0.0858	0.8837	0.1163	0.2049	0.0772
						C16 Evaluation formats	0.1296	0.7949	0.2051	0.3613	0.2057

#### 3.2.4 Result analysis

The results showed that, at the system level, the weight of B1 course philosophy (0.3801) was the highest, followed by B4 course evaluation (0.3239), B3 Course Teaching (0.2041), and B2 course content (0.0919). This finding is consistent with Tyler's (2014) view that once educational objectives are established, course philosophy plays a leading role in curriculum construction. Course philosophy serves as the criterion for selecting course evaluation, teaching methods, and course content.

At the level of the criteria layer, B1 course philosophy had the greatest weight. This is consistent with Tyler's curriculum theory, which holds that course philosophy is the core of curricular activity. It has a governing function over course teaching, course content, and course evaluation. The result suggests that the current course philosophy still largely extends traditional assumptions and has not yet undergone a comprehensive transformation. Simultaneously, consensus regarding constructivist philosophy and the student-centered principle has clearly increased, which is highly consistent with recent research on paradigm shifts in design pedagogy (Meyer and Norman, 2020).

B4 course evaluation ranked second. Course evaluation is the methodological and technical means through which course philosophy is enacted, and course objectives are realized, and it also reflects the alignment between evaluation and objectives. This understanding represents an upgrading of traditional course evaluation and reflects the broader transformation toward future learning paradigms. It also highlights the role of evaluation as the central hub of curriculum implementation in modern educational thought. This weighting outcome not only underscores the methodological innovation of course evaluation, but also signals its transformation from a tool for verifying outcomes into an integrated support system linking objectives, processes, and development. This finding supports Carless's (2015) argument that evaluation functions as a learning mechanism.

B3 course teaching ranked third, indicating a significant shift away from the traditional emphasis on teaching and toward evaluation. In the context of increasingly interdisciplinary knowledge in design disciplines, course evaluation can serve as a lever for course implementation and generate synergy between evaluation and teaching, thereby providing targeted support for achieving course objectives.

The B2 course content had the lowest weight. This does not diminish its importance; rather, it reflects that under sustainable and interdisciplinary educational principles, course content has shifted from a fixed knowledge base to a dynamic toolkit, with its systemic function relying primarily on the philosophical guidance and evaluation. Especially as design education is currently transitioning from skill transmission to creativity development, course content, as the traditional carrier of skill training, such as hand drawing and software operation, was strategically assigned a lower weight to highlight newly emerging dimensions relevant to creativity development in Design Disciplines.

**Figure 5:**
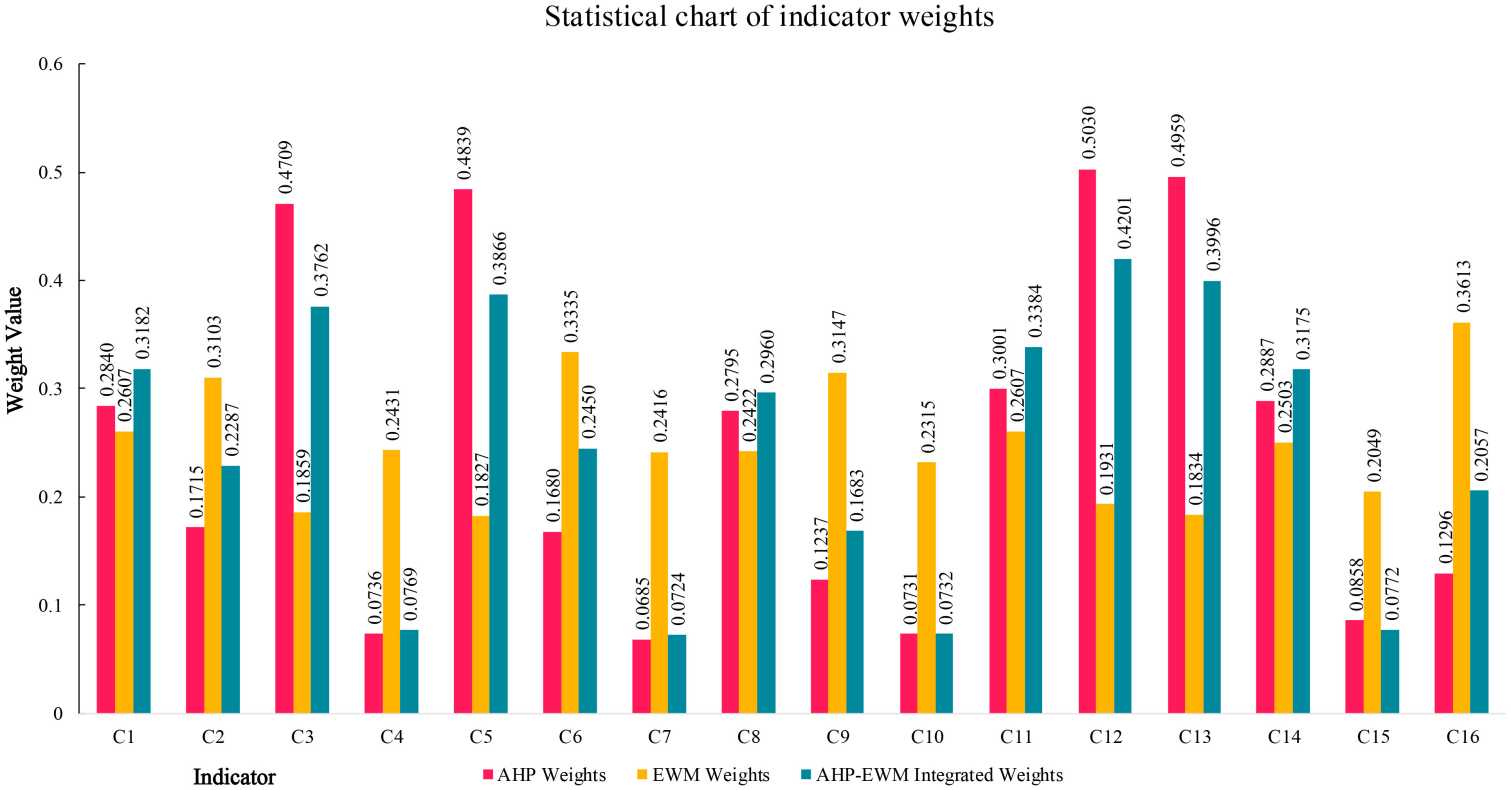
Composite weight values of the indicators.

Ranking the second-level indicators from highest to lowest weight yielded the following order: C12 application of inquiry-based teaching > C13 formative assessment > C5 creativity literacy > C3 sustainability > C11 application of teaching strategies > C1 constructivist philosophy > C14 performance assessment > C8 interdisciplinary literacy > C6 critical thinking > C2 student-centered principle > C16 evaluation formats > C9 pre-course cognitive diagnostic assessment > C15 evaluation agents > C4 learning environment > C10 application of learning models > C7 practical ability. These indicators reflect the supporting roles of different dimensions in relation to the core objective of creativity development and represent important factors for evaluating ESD-oriented creativity courses in design disciplines, as well as key issues that should be addressed during course implementation (Figure 5).

## 4 Fuzzy comprehensive evaluation

### 4.1 Method of fuzzy comprehensive evaluation

Fuzzy comprehensive evaluation (FCE) is a method based on the principles of fuzzy mathematics that transforms qualitative evaluation into quantitative evaluation by calculating the membership degree of each indicator to the evaluation objectives (Yanwen et al., 2022). It allows comparison of numerous factors and indicators that are constrained by various elements and are difficult to quantify during the evaluation process, thereby forming a clearer overall assessment. After determining indicator weights through the AHP-EWM combined method, the steps for conducting FCE are as follows:

(1) Determine the factor set of the evaluation object.

Let *U* = (*u*_1_, *u*_2_, ⋯*u*_*m*_) be the collection of various indicators that can characterize the features of the evaluation objective. In this study, creativity courses in the design discipline are evaluated from four aspects: course philosophy, course content, course teaching, and course evaluation. Thus, the factor set is *U* = *{course philosophy, course content, course teaching, course evaluation}*.

(2) Determine the comment set.

Use *V* = (*v*_1_, *v*_2_, ⋯*v*_*m*_) to represent the evaluation grades for various indicators that characterize the evaluation objective, typically divided into 3–5 levels. In this study, the evaluation grades are divided into 5 levels, with each level corresponding to a fuzzy subset.

(3) Establish the overall fuzzy membership matrix R.

The fuzzy membership matrix R is constructed for all evaluation indicators and evaluated objectives as follows (Equation 14):


$$\begin{array}{c} R={\left(r_{ij}\right)}_{m\times n}=\left[\begin{array}{cccc} r_{11} & r_{12} & \cdots & r_{1n}\\ r_{21} & r_{22} & \cdots & r_{2n}\\ \vdots & \vdots & \ddots & \vdots \\ r_{m1} & r_{m2} & \cdots & r_{mn} \end{array}\right] \end{array}$$ (14)

(4) Determine the weight vector of evaluation factors.

Assume the set of weight vectors for indicators is *W* = (*w*_1_, *w*_2_, ⋯*w*_*m*_). In this set, *w*_*i*_ represents the weight of the indicator in the fuzzy subset *u*_*i*_. In this study, the weights were obtained using AHP and then normalized. The calculation method is as follows (Equation 15):


$$\begin{array}{c} \overset{m}{\underset{i=1}{\sum }}w_{i}=1,\;w_{i}\geq 0,\;i=1,2,\cdots m \end{array}$$ (15)

(5) Synthesize the fuzzy comprehensive evaluation vector.

Perform matrix synthesis operation (Equation 16):


$$\begin{array}{c} B=\left(w_{1},w_{2},\cdots w_{m}\right)\left[\begin{array}{cccc} r_{11} & r_{12} & \cdots & r_{1n}\\ r_{21} & r_{22} & \cdots & r_{2n}\\ \vdots & \vdots & \ddots & \vdots \\ r_{m1} & r_{m2} & \cdots & r_{mn} \end{array}\right]=\left(b_{1},b_{2},\cdots b_{n}\right) \end{array}$$ (16)

(6) Analyze the fuzzy comprehensive evaluation results.

When dealing with practical problems, the principle of maximum membership degree is often applied to analyze the calculation results of fuzzy comprehensive evaluation. However, due to the subjective nature of data collection during model construction, the calculation results may sometimes be unreasonable. To address this, the weighted average method is chosen to quantify the final evaluation results by seeking the membership grade (Equation 17).


$$\begin{array}{c} F=VB^{T}=\left(v_{1},v_{2},\cdots v_{n}\right)\left(\begin{array}{c} b_{1}\\ b_{2}\\ \vdots \\ b_{n} \end{array}\right) \end{array}$$ (17)

In the comprehensive evaluation of this study, five levels of comments are set for each indicator, namely *V* = *[V*_1_*, V*_2_*, V*_3_*, V*_4_*, V*_5_*]* = *[Excellent, Good, Average, Poor, Very Poor]*, and assigned scores are *V* = *[100, 80, 60, 40, 20]*. The evaluation set for each indicator must be clearly defined, with qualitative indicators described in clear language and quantitative indicators assigned corresponding reference values to determine their respective levels, thereby enabling the transformation from qualitative to quantitative.

### 4.2 Empirical analysis of fuzzy comprehensive evaluation method (FCE)

Because the evaluation of sustainable creativity courses in design disciplines involves multiple dimensions, considerable complexity, and a clear hierarchical structure, this study constructed a multi-level FCE indicator system and adopted a two-level FCE for empirical analysis. The evaluation indicator system had already been weighted using combined weighting (AHP-EWM), ensuring the scientific rigor and objectivity of the weight distribution. To examine the feasibility and preliminary application effect of the proposed evaluation model, a representative case was selected for exploratory analysis.

Regional universities are an important component of China's higher education system and generally offer programs related to design disciplines. They therefore have a far-reaching influence on the overall improvement of creativity among design majors in China. This study selected the School of Art and Design at “H” University, a regional university in Anhui Province, as the research site and chose the course on innovative design methods in the product design program for case analysis. To scientifically evaluate the teaching effectiveness of creativity courses in design disciplines at this university, this study adopted FCE. It combined it with a two-level FCE to achieve a systematic assessment across multiple levels and factors. First, the established evaluation indicators were transformed into questionnaire descriptions (Table 10). Second, to ensure the authenticity and depth of the empirical data, the research team used purposive sampling to select 20 core stakeholders who were deeply involved in the course as Evaluation Agents. The participants included 12 teachers, three teaching administrators, and five students. All participants had taken part in classroom teaching. Apart from the students, all had substantial course experience and were familiar with the course development goals. The data collection process strictly followed academic ethics. Face-to-face interview-style questionnaires were used to ensure that evaluators fully understood the meaning of each indicator, thereby minimizing arbitrary subjectivity as much as possible. All participants evaluated each indicator in the system on a five-level scale (very good, good, average, poor, very poor) and assigned the corresponding score values (100/80/60/40/20).

**Table 10:** Questionnaire descriptions for evaluation indicator.

Description of indicators	Descriptions
C1 Constructivist philosophy	What do you think of the importance of constructivist learning principles for cultivating student creativity?
C2 Student-centered principle	What do you think of the role of student-centered course design in enhancing learning initiative?
C3 Sustainability	What do you think of the necessity of integrating sustainability values into the Course Philosophy?
C4 Learning environment	What do you think of the impact of collaborative and exploratory learning environments on creativity stimulation?
C5 Creativity literacy	What do you think of the centrality of Creativity Literacy (novelty, functionality, and systematicity) in course objectives?
C6 Critical thinking	What do you think of the necessity of systematically developing Critical Thinking?
C7 Practical ability	What do you think of the significance of project-based learning for solving real-world problems?
C8 Interdisciplinary literacy	What do you think of the value of integrating Interdisciplinary Literacy (STEM, arts, and society) into course design?
C9 Pre-course cognitive diagnostic assessment	What do you think of the utility of pre-course cognitive diagnostics for precision teaching?
C10 Application of learning models	What do you think of the effectiveness of flexibly adapting different learning models (deep, strategic, and surface)?
C11 Application of teaching strategies	What do you think of the effectiveness of differentiated instruction and collaborative learning in enhancing creativity?
C12 Application of inquiry-based teaching	What do you think of the critical role of inquiry-based or problem-based teaching in fostering innovative thinking?
C13 Formative assessment	What do you think of the impact of continuous formative feedback on learning progression?
C14 Performance assessment	What do you think of the validity of performance-based evaluation (e.g., portfolios and exhibitions) for measuring creativity?
C15 Evaluation agents	What do you think of the necessity of involving multiple stakeholders (faculty, students, and industry) in evaluation?
C16 Evaluation formats	What do you think of the importance of multiple evaluation formats (e.g., self-assessment, peer assessment, and digital assessment)?

Experts were then invited to rate each indicator in the indicator layer independently. By summarizing the number of ratings each indicator received across the grade levels, the membership degree of that indicator to a given evaluation grade could be obtained. The proportion of the 20 evaluators who selected a given grade for each indicator was treated as the membership degree (Table 11), thereby establishing the single-factor fuzzy evaluation matrix. Under the AHP-EWM weighting scheme, the final comprehensive course quality score was 70.35, corresponding to an average level. Among the four dimensions, course evaluation received the highest score (76.5037), whereas course philosophy received the lowest score (62.7816). The calculation process is shown below.

**Table 11:** Survey table of fuzzy membership degrees for the evaluation indicators of ESD-oriented creativity courses in design disciplines.

Evaluation objective	Factor set *U_*i*_*		Evaluation set *V_*i*_*				
			*V*_1_ (excellent)	*V*_2_ (good)	*V*_3_ (average)	*V*_4_ (poor)	*V*_5_ (very poor)
A ESD-oriented creativity course objectives in design disciplines	B1 Course philosophy	C1 Constructivist philosophy	1	6	9	4	0
		C2 Student-centered principle	0	3	12	5	0
		C3 Sustainability	1	7	8	3	1
		C4 Learning environment	2	6	9	2	1
	B2 Course content	C5 Creativity literacy	6	6	6	2	0
		C6 Critical thinking	1	9	9	1	0
		C7 Practical ability	6	7	7	0	0
		C8 Interdisciplinary literacy	2	9	9	0	0
	B3 Course teaching	C9 Pre-course cognitive diagnostic assessment	3	7	9	1	0
		C10 Application of learning models	3	9	6	1	1
		C11 Application of teaching strategies	3	7	7	2	1
		C12 Application of inquiry-based teaching	5	9	4	2	0
	B4 Course evaluation	C13 Formative assessment	8	7	4	1	0
		C14 Performance assessment	4	9	7	0	0
		C15 Evaluation agents	4	9	5	1	1
		C16 Evaluation formats	0	10	7	2	1

(1) Calculate the evaluation vector for the B1 course philosophy:

$$\begin{array}{l} B_{1}=\left(0.3182,0.2287,0.3762,0.0769\right)\\ \left[\begin{array}{rrrrr} 0.0500 & 0.3000 & 0.4500 & 0.2000 & 0.0000\\ 0.0000 & 0.1500 & 0.6000 & 0.2500 & 0.0000\\ 0.0500 & 0.3500 & 0.4000 & 0.1500 & 0.0500\\ 0.1000 & 0.3000 & 0.4500 & 0.1000 & 0.0500 \end{array}\right]\\ \text{=}(0.0424,0.2845,0.4655,0.1849,0.0227) \end{array}$$

(2) Calculate the evaluation vector for the B2 course content:

$$\begin{array}{c} B_{2}=\left(0.3866,0.2450,0.0724,0.2960\right)\\ \left[\begin{array}{ccccc} 0.3000 & 0.3000 & 0.3000 & 0.1000 & 0.0000\\ 0.0500 & 0.4500 & 0.4500 & 0.0500 & 0.0000\\ 0.3000 & 0.3500 & 0.3500 & 0.0000 & 0.0000\\ 0.1000 & 0.4500 & 0.4500 & 0.0000 & 0.0000 \end{array}\right]\\ \text{=}\left(0.1796,0.3848,0.3848,0.0509,0.0000\right) \end{array}$$

(3) Calculate the evaluation vector for the B3 course teaching:

$$\begin{array}{c} B_{3}=\left(0.1683,0.0732,0.3384,0.4201\right)\\ \left[\begin{array}{ccccc} 0.1500 & 0.3500 & 0.4500 & 0.0500 & 0.0000\\ 0.1500 & 0.4500 & 0.3000 & 0.0500 & 0.0500\\ 0.1500 & 0.3500 & 0.3500 & 0.1000 & 0.0500\\ 0.2500 & 0.4500 & 0.2000 & 0.1000 & 0.0000 \end{array}\right]\\ \text{=}\left(0.1920,0.3993,0.3002,0.0879,0.0206\right) \end{array}$$

(4) Calculate the evaluation vector for the B4 course evaluation:

$$\begin{array}{c} B_{4}=\left(0.3996,0.3175,0.0772,0.2057\right)\\ \left[\begin{array}{ccccc} 0.4000 & 0.3500 & 0.2000 & 0.0500 & 0.0000\\ 0.2000 & 0.4500 & 0.3500 & 0.0000 & 0.0000\\ 0.2000 & 0.4500 & 0.2500 & 0.0500 & 0.0500\\ 0.0000 & 0.5000 & 0.3500 & 0.1000 & 0.0500 \end{array}\right]\\ \text{=}\left(0.2388,0.4203,0.2823,0.0444,0.0141\right) \end{array}$$

Consequently, the fuzzy membership matrix for the primary indicators can be obtained as:


$$\begin{array}{c} R=\left[\begin{array}{ccccc} 0.0424 & 0.2845 & 0.4655 & 0.1849 & 0.0227\\ 0.1796 & 0.3848 & 0.3848 & 0.0509 & 0.0000\\ 0.1920 & 0.3993 & 0.3002 & 0.0879 & 0.0206\\ 0.2388 & 0.4203 & 0.2823 & 0.0444 & 0.0141 \end{array}\right] \end{array}$$

The weight vector for the primary indicators, previously derived using AHP-EWM, is:


$$\begin{array}{c} W=\left(0.3801,0.0919,0.2041,0.3239\right) \end{array}$$

Multiplying the weights of the primary indicators by their fuzzy membership matrix yields the overall evaluation vector:


$$\begin{array}{c} B=WR=\left(0.1492,0.3611,0.3650,0.1073,0.0174\right) \end{array}$$

Based on the overall evaluation vector and the grade score vector, the overall score is calculated (see Table 12).

**Table 12:** Standards for the overall score of the evaluation object.

Evaluation level	*V*_1_ (excellent)	*V*_2_ (good)	*V*_3_ (average)	*V*_4_ (poor)	*V*_5_ (very poor)
Score	100	80	60	40	20

Using *F*=*VB*^*T*^ *to* calculate the evaluation score. The calculated overall score *F* is:


$$\begin{array}{c} F=VB^{T}=\left(100\;80\;60\;40\;20\right)\left[\begin{array}{c} 0.1492\\ 0.3611\\ 0.3650\\ 0.1073\\ 0.0174 \end{array}\right]=70.3472 \end{array}$$

As shown in Table 13, the differences in evaluation values across the first-level indicators were substantial, yielding the rank order B4 course evaluation > B2 course content > B3 course teaching > B1 course philosophy. This result reveals a clear imbalance and reflects a structural misalignment in the transformation of design education at regional universities.

**Table 13:** Evaluation results for each indicator of the creativity courses in design disciplines.

Target layer	Evaluation value	Alternative layer	Evaluation value	Criteria layer	Evaluation value	Rank
A ESD-oriented creativity course objectives in design disciplines	70.3472	B1 Course philosophy	62.7816	C1 Constructivist philosophy	64.0000	15
				C2 Student-centered principle	58.0000	16
				C3 Sustainability	64.0000	14
				C4 Learning environment	66.0000	13
		B2 Course content	73.8592	C5 Creativity literacy	76.0000	5
				C6 Critical thinking	70.0000	11
				C7 Practical ability	79.0000	2
				C8 Interdisciplinary literacy	73.0000	8
		B3 Course teaching	73.0853	C9 Pre-course cognitive diagnostic assessment	72.0000	9
				C10 Application of learning models	72.0000	10
				C11 Application of teaching strategies	69.0000	12
				C12 Application of inquiry-based teaching	77.0000	3
		B4 Course evaluation	76.5037	C13 Formative assessment	82.0000	1
				C14 Performance assessment	77.0000	4
				C15 Evaluation agents	74.0000	7
				C16 Evaluation formats	66.0000	14

Among the four dimensions, the B1 course philosophy obtained a score of 62.7816, the lowest of all. Compared with the model result in which B1 had the highest weight (0.3801), this phenomenon of a high weight but a low score clearly indicates that the course philosophy of design disciplines at this university remains relatively conservative and largely operates within a traditional teaching model centered on skill transmission. This suggests that foundational educational principles such as constructivist philosophy and sustainability values have not yet been deeply internalized in actual teaching practice. Nor do they fully reflect the interdisciplinary character and systemic creativity development goals required in the AI era. Specifically, the current philosophy is still not fully aligned with the developmental needs of future society.

B3 course teaching (73.0853) and B2 course content (73.8592) obtained similar scores, ranking third and second, respectively. This indicates that the implementation of creativity courses in design disciplines at the university still relies heavily on an existing knowledge system, emphasizes traditional methods of course delivery, and foregrounds the central role of teachers and content. Overall performance in these dimensions was relatively stable, but lacked substantial breakthroughs.

Notably, the B4 course evaluation (76.5037) achieved the highest score among the four dimensions. This indicates that course evaluation already plays a significant regulatory role in the current course implementation process and that participants have recognized its guiding function. Simultaneously, however, it reveals a typical contradiction in the educational transformation of creativity courses in design disciplines at this university, namely, the precedence of the instrumental rationality of evaluation over the value rationality of course philosophy. Teachers have already made some attempts at the technical level of evaluation practice, such as using formative assessment tools but the deeper values that govern teaching remain constrained by traditional paradigms. This phenomenon verifies the importance of the evaluation model constructed in this study. It is not merely a measurement tool but also a precise diagnostic tool capable of identifying the point at which a disconnect occurs in implementing educational philosophy during educational transformation.

**Figure 6:**
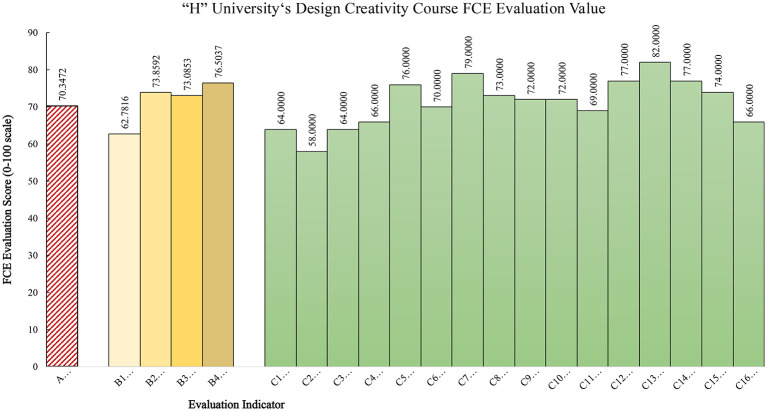
FCE evaluation values.

Overall, based on the AHP-EWM-FCE model, the total evaluation score of the creativity courses in design disciplines at “H” University was 70.3472, indicating an average level (Figure 6). In response to this result, future improvement should use B4 Course Evaluation as a lever and introduce strategies such as constructivist philosophy, sustainability principles, and interdisciplinary construction to promote a transition in course philosophy from conservative inheritance to innovation-driven development, thereby ultimately achieving a reconstruction of the educational paradigm of creativity courses in design disciplines.

## 5 Conclusions

This study integrated the AHP-EWM and FCE to construct and empirically examine a course evaluation system for creativity courses in design disciplines oriented to ESD. In doing so, it addressed the three major goals proposed at the outset and validated both the scientific rigor and practical value of the model. The core findings can be summarized in three aspects.

### 5.1 A four-dimensional evaluation framework integrating ESD and creativity development was established, and course philosophy and course evaluation were identified as the two key leverage points

This study first identified four core dimensions for evaluating ESD-oriented creativity courses in design disciplines: course philosophy, course content, course teaching, and course evaluation, which were further refined into 16 specific indicators. Using combined weighting (AHP-EWM), the study found that the weights of course philosophy (0.3801) and course evaluation (0.3239) were substantially higher than those of the other dimensions, indicating that they are the two most critical factors influencing the effectiveness of creativity development. Among the secondary indicators, application of inquiry-based teaching (C12) and formative assessment (C13) received the highest weights, highlighting the importance of student-centered pedagogical and evaluative orientations that foster innovative thinking through active inquiry and process feedback. This weight structure suggests that the primary focus for advancing the transformation of design education toward ESD and creativity lies in renewing course philosophy and reconstructing evaluation paradigms.

### 5.2 A methodological combination was provided to address the problems of evaluative subjectivity and generalized indicators

To address the strong subjectivity and static generalization of indicators in traditional Course Evaluation, this study innovatively adopted an integrated methodological approach that combines AHP-EWM and FCE. The integration of AHP and EWM created a balance between expert value judgments and objective data variation, so that the indicator weights reflected both educational value orientation and actual data distribution. This enhanced the robustness and persuasiveness of the weighting system. Subsequently, the FCE model transformed qualitative descriptions, such as the manifestation of critical thinking, into quantitative membership degrees, thereby enabling a multidimensional, multilevel quantitative evaluation of course quality. This methodological combination provides a replicable and operational technical solution for evaluating complex literacy in design education and other practice-oriented disciplines.

### 5.3 The empirical assessment revealed deep contradictions in the transformation of Design Education at regional universities and identified feasible pathways for improvement

The FCE-based empirical assessment of a regional university showed that the overall course score was 70.35, corresponding to an average level, and that the internal structure displayed a marked split between high evaluation and low philosophy. The course evaluation dimension received the highest score (76.50), whereas the course philosophy dimension received the lowest score (62.78). This sharply reveals the structural dilemma of the current transformation process, namely, the precedence of instrumental rationality and the lag of value rationality. Evaluation methods may have been updated, but the deeper philosophy guiding teaching remains trapped in a traditional skill-training paradigm, preventing core principles such as sustainability and constructivist philosophy from being effectively implemented. This finding confirms existing criticism of the teacher-centered tendency in design education and indicates clearly that future reform must go beyond the introduction of isolated techniques or methods. Instead, evaluation reform should be used as a lever to trigger the systematic reconstruction of course philosophy and teaching culture, thereby enabling a genuine paradigm shift toward the development of creativity and sustainability literacy.

## 6 Discussion

By constructing the evaluation model and analyzing indicator weights, this study found that course philosophy (weight = 0.3801) and course evaluation (weight = 0.3239) are the two core dimensions influencing creativity development in design disciplines. This weighting structure reveals an important paradigm shift: the traditional linear instructional process of “objectives-content-process-evaluation” is being reversed into a design logic of “philosophy-evaluation-teaching-content.” This transformation has profound implications for design education. As Wiggins and McTighe (2005) argued in their concept of backward design, placing evaluation before instructional activities can ensure consistency between educational objectives and outcomes. In the present study, the high weights assigned to course philosophy and course evaluation further indicate that the transformation of design education cannot be limited to updating content alone. Rather, it must rely on evaluation mechanisms to drive the reconstruction of teaching approaches and course philosophy, which closely aligns with the principle of constructive alignment emphasized by Hristov et al. (2023).

The empirical analysis further revealed a concrete pathway through which evaluation can promote reform in Design Education, but the assessment results of the case university also displayed a marked contradiction between high evaluation and low philosophy. Although the course evaluation dimension received the highest score (76.50), the dimensions related to sustainability and constructivist philosophy, which should constitute the core of course philosophy, received the lowest score (62.78). Moreover, key philosophical indicators such as sustainability (C3) and constructivist philosophy (C1) both obtained actual evaluation values of only 64, far below the level of importance suggested by their weights. This contrast indicates that current course practice remains deeply embedded in technical rationality and has failed to fully integrate ecological symbiosis thinking into the development of design creativity. At a deeper level, this disconnect between instrumental rationality and value rationality confirms Saris's (2020) criticism of the teacher-centered tendency in Chinese design education and also highlights the common challenges of internalizing ESD principles when they are implemented in micro-level disciplinary contexts (Wals, 2022). Therefore, future reform must use evaluation as a lever and shift the course philosophy from a conservative inheritance to innovation-driven development.

### 6.1 Theoretical contributions of this study

The evaluation system developed in this study not only addresses practical needs but also fills important gaps in three academic fields.

First, in the theory of creativity evaluation in design education, existing assessments have tended to focus on the formal novelty and technical feasibility of works and have thus suffered from the limitation of emphasizing skills while neglecting thinking, with little alignment to ESD principles. This study moves beyond that framework by incorporating ecological ethics and interdisciplinary thinking into the evaluation system. In doing so, it addresses the absence of sustainability literacy in traditional evaluation (Boehnert et al., 2022) and provides a new quantitative evaluation tool for assessing responsible design innovation (Boehnert et al., 2022).

Second, regarding the tool-oriented development of ESD evaluation, this study transitions from qualitative description to integrated qualitative-quantitative assessment. Previous studies often remained at the level of conceptual discussion and lacked discipline-adapted quantitative models (Annan-Diab and Molinari, 2017). By constructing a 16-indicator system, this study provides a replicable micro-level evaluation tool for implementing the VAA triad.

Third, this study contributes methodological innovation to educational evaluation. On the one hand, the integration of AHP and EWM reconciles differences between value judgments and objective data, thereby alleviating the subjective bias associated with single weighting methods (Yang et al., 2026). Conversely, this study advances a fundamental shift in evaluation paradigms, moving from Tyler's (2014) classical linear model to a reverse design paradigm. This new orientation is better suited to student-centered, process-oriented educational demands and offers a new model for evaluating similar courses.

### 6.2 Contemporary context and future significance

These theoretical contributions are particularly necessary in the current era. In the age of AI, although Gen AI can assist with idea generation, human critical thinking and systemic innovative ability remain the core competitive strengths. The key roles of inquiry-based teaching and formative assessment revealed in this study point directly to a pathway through which design education can move from skill transmission to the development of creativity literacy. Using AI-assisted real-time feedback, it may become possible to further optimize students' learning pathways.

Furthermore, this study is consistent with China's strategy to develop the emerging liberal arts. The Emerging Liberal Arts emphasizes interdisciplinary integration and value reconstruction, and the evaluation framework developed here constitutes a concrete response within design disciplines to the objective of cultivating compound talents. This indicates that the transformation of design education is not only a disciplinary necessity but also a crucial means of responding to national strategy and cultivating innovative talent capable of addressing future complex challenges.

## 7 Prospects and limitations

This study constructed and preliminarily validated an evaluation model for creativity courses in design disciplines oriented to ESD, but it still has certain limitations in both depth and breadth. First, it should be emphasized that, in terms of sample size and generalizability, this study is essentially an exploratory case study. The empirical validation was based on a single course at one regional university in China (“H” University). Although this case is somewhat representative of the current state of Design Education in local application-oriented higher education institutions in China, and although the primary purpose of the study was to validate the internal logic of model construction, the broader generalizability of the conclusions still requires further examination. As noted in the discussion, the applicability of this model may be affected by differences in institutional type, regional culture, and disciplinary orientation. For example, research universities and vocational institutions place very different emphases on talent cultivation objectives.

Second, there is still room for improvement in expert consultation and data collection. Although the evaluation system was developed through multiple rounds of discussion with 16 experts, the breadth of the expert sample in terms of disciplinary background and geographical distribution could be expanded further. Simultaneously, the FCE data came from 20 course-related participants. Although the evaluations were based on authentic teaching experiences, the influence of individual subjective perspectives cannot be completely ruled out. Furthermore, from a methodological standpoint, although combined weighting (AHP-EWM) was adopted to integrate subjective and objective information, human subjective judgment still cannot be entirely eliminated in steps such as indicator screening and judgment matrix construction. This is also an inherent challenge of such mixed-method approaches.

In light of these limitations, future research may be deepened in several directions. At the horizontal level, the evaluation framework can be applied to a wider range of contexts. For example, empirical data from different regions and types of universities can be compared to dynamically calibrate indicator weights, thereby improving the model's multi-university adaptability. At the longitudinal level, qualitative methods such as in-depth interviews and long-term classroom observation should be combined to investigate the deeper causes of structural contradictions, such as high evaluation but low philosophy, including institutional constraints and resource support, to reveal the dynamic mechanisms involved in the transformation of design education. Finally, as AI grows, it will also be important to explore how learning analytics and GenAI can be integrated into the evaluation process. For example, AI could be used to analyze thinking trajectories reflected in design drafts, thereby enabling real-time collection and feedback of evaluation data and further enhancing the intelligence and efficiency of the evaluation model.

## Data Availability

The original contributions presented in the study are included in the article/Supplementary material, further inquiries can be directed to the corresponding author.
