# Supplementary material for: Developing an evaluation system for creativity courses in design disciplines oriented to education for sustainable development: an integrated application of AHP-entropy weighting and FCE models
Source: Front Psychol. 2026 Apr 30;17:1709780. doi: 10.3389/fpsyg.2026.1709780 (PMC13171502; doi:10.3389/fpsyg.2026.1709780)
Supplement: Supplementary file 1 [file Data_Sheet_1.PDF]

Supplementary Table S1

Table 6. Judgment Matrix for the Criteria Layer

	B1 Course Philosophy	B2 Course Content	B3 Course Teaching	B4 Course Evaluation
B1 Course Philosophy	1	4	3	2
B2 Course Content	1/4	1	1/2	1/3
B3 Course Teaching	1/3	2	1	1/2
B4 Course Evaluation	1/2	3	2	1

Judgment Matrix for the Alternative Layer

	C1 Constructivist Philosophy	C2 Student-Centred Principle	C3 Sustainability	C4 Learning Environment
C1 Constructivist Philosophy	1	2	1/2	4
C2 Student-Centred Principle	1/2	1	1/3	3
C3 Sustainability	2	3	1	5
C4 Learning Environment	1/4	1/3	1/5	1

	C5 Creativity Literacy	C6 Critical Thinking	C7 Practical Ability	C8 Interdisciplinary Literacy
C5 Creativity Literacy	1	3	6	2
C6 Critical Thinking	1/3	1	3	1/2
C7 Practical Ability	1/6	1/3	1	1/4
C8 Interdisciplinary Literacy	1/2	2	4	1

	C9 Pre-course Cognitive Diagnostic Assessment	C10 Application of Learning Models	C11 Application of Teaching Strategies	C12 Application of Inquiry-Based Teaching
C9 Pre-course Cognitive Diagnostic Assessment	1	2	1/3	1/4
C10 Application of Learning Models	1/2	1	1/4	1/6
C11 Application of Teaching Strategies	3	4	1	1/2
C12 Application of Inquiry-Based Teaching	4	6	2	1

	C13 Formative Assessment	C14 Performance Assessment	C15 Evaluation Agents	C16 Evaluation Formats
C13 Formative Assessment	1	2	5	4
C14 Performance Assessment	1/2	1	3	3
C15 Evaluation Agents	1/5	1/3	1	1/2
C16 Evaluation Formats	1/4	1/3	2	1
